# Synthetic aporphine alkaloids are potential therapeutics for Leigh syndrome

**DOI:** 10.1038/s41598-024-62445-w

**Published:** 2024-05-21

**Authors:** Mizuki Kobayashi, Akihiko Miyauchi, Eriko F. Jimbo, Natsumi Oishi, Shiho Aoki, Miyuki Watanabe, Yasushi Yoshikawa, Yutaka Akiyama, Takanori Yamagata, Hitoshi Osaka

**Affiliations:** 1 Department of Pediatrics, Division of Pediatrics, Jichi Medical University, 3311-1 Yakushiji, Shimotsuke, Tochigi 329-0498 Japan; 2 School of Life Science and Technology, Tokyo Institute of Technology, Yokohama, 226-8501 Japan; 3 Department of Computer Science, School of Computing, Tokyo Institute of Technology, Meguro-ku, Tokyo, 152-8550 Japan; 4 Middle-Molecule IT-Based Drug Discovery Laboratory (MIDL), Tokyo Institute of Technology, Kawasaki, Kanagawa 210-0821 Japan

**Keywords:** Biochemistry, Drug discovery, Diseases, Medical research

## Abstract

Mitochondrial diseases are mainly caused by dysfunction of mitochondrial respiratory chain complexes and have a variety of genetic variants or phenotypes. There are only a few approved treatments, and fundamental therapies are yet to be developed. Leigh syndrome (LS) is the most severe type of progressive encephalopathy. We previously reported that apomorphine, an anti- “off” agent for Parkinson’s disease, has cell-protective activity in patient-derived skin fibroblasts in addition to strong dopamine agonist effect. We obtained 26 apomorphine analogs, synthesized 20 apomorphine derivatives, and determined their anti-cell death effect, dopamine agonist activity, and effects on the mitochondrial function. We found three novel apomorphine derivatives with an active hydroxy group at position 11 of the aporphine framework, with a high anti-cell death effect without emetic dopamine agonist activity. These synthetic aporphine alkaloids are potent therapeutics for mitochondrial diseases without emetic side effects and have the potential to overcome the low bioavailability of apomorphine. Moreover, they have high anti-ferroptotic activity and therefore have potential as a therapeutic agent for diseases related to ferroptosis.

## Introduction

Mitochondrial diseases are caused by pathogenic variants of either nuclear or mitochondrial DNA, resulting in mitochondrial respiratory chain complex dysfunction or morphological abnormalities^1,2^. Mitochondrial dysfunction leads to decreased ATP production and oxidative stress, resulting in cell death and a loss of function in affected organs. Among the various phenotypes of mitochondrial diseases, mitochondrial myopathy, encephalopathy, lactic acidosis, stroke-like episodes (MELAS), Kearns-Sayre syndrome (KSS), and Leigh syndrome (LS) are the three major clinical types of mitochondrial diseases^3^.

LS is one of the most severe types of mitochondrial disease, and its onset occurs during childhood. Patients with LS show progressive developmental regression along with bilateral central nervous system (CNS) lesions, especially in the basal ganglia and brain stem. Basal ganglia and brain stem symptoms include hypertonia, involuntary movements, hearing loss, dystonia, and respiratory interruption. Epilepsy, cerebellar symptoms and hypotonia may also occur. LS is often diagnosed after an acute exacerbation triggered by infections episodes, resulting in rapid metabolic failure due to an energy crisis. Systemic symptoms may include hepatic and myocardial dysfunction^4^. An estimated 20–30% of LS infants with an early onset (as young as 6 months after birth) experience progressive regression in both the motor and intellectual functions and 80% mortality by 10 years old^4,5^. Although medications such as idebenone for Leber hereditary optic neuropathy (LHON) and taurine for MELAS with the m.3243A > G variant have been approved for mitochondrial diseases, LS patients are treated with supportive therapy of vitamins and OXPHOS complex cofactors. No causative treatment has been established for LS to date, and the development of a targeted therapy is awaited^1,6^.

Apomorphine has been used as a dopamine receptor agonist to treat advanced and drug-resistant Parkinson’s disease (PD) “off” symptoms. In addition, the antioxidant, and neuroprotective effects of apomorphine and its efficacy in non-motor symptoms in patients with PD have been repeatedly reported^7,8^. In a previous study, apomorphine was screened from a CNS drug library and evaluated for its anti-cell death effect in L-buthionine-(S, R)-sulfoximine (BSO)-loaded LS patient-derived skin fibroblasts^9^. This suggests the possibility of repositioning apomorphine as a therapeutic agent for mitochondrial diseases. Furthermore, it has also been reported that apomorphine has anti-ferroptotic effects independent of dopamine agonist activity^10^.

The challenges in repositioning apomorphine as a mitochondrial disease drug are its strong dopamine D2 receptor agonist (DA) effect and short biological half-life. The DA effect was originally demonstrated in PD, but in this case, it led to the side effect of emesis. The biological half-life of apomorphine, as short as 30 min, makes it undesirable as a mitochondrial disease drug^11,12^. In addition, apomorphine is not suitable for oral administration because of its low bioavailability of less than 4% for first-pass effects, so various other administration routes have been investigated.

In the present study, we obtained 20 apomorphine analogs and synthesized 26 derivatives based on the aporphine framework. We then screened their DA activity and anti-cell death effect under oxidative stress to obtain compounds that retain anti-cell death effects similar or superior apomorphine but lack DA activity.

## Results

### Obtaining 26 apomorphine analogs

**Figure 1:**
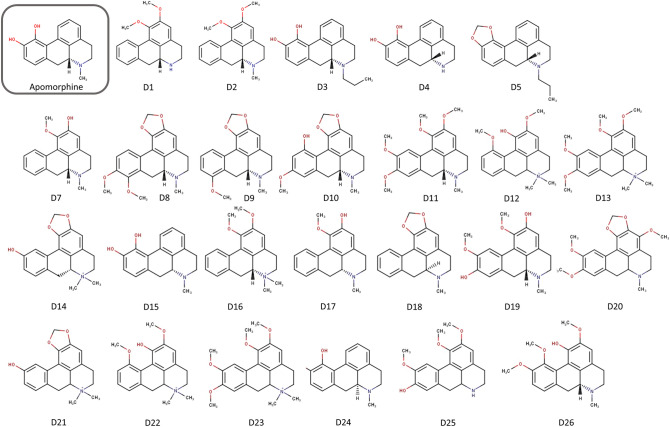
Structural formula of 26 apomorphine analogs. We obtained 26 apomorphine analogs from 2,000,000 compounds through screening by structural similarity calculation. D1 is nuciferin, and D15 is apomorphine. D6 is vacant.

Figure 1 shows the structural formulas of the 26 apomorphine analogs obtained by structural similarity calculation. International Union of Pure and Applied Chemistry (IUPAC) names are shown in Supplementary Table S1. The compounds were numerically labeled in the order of acquisition after heading D. Some of them have nonproprietary names; D1 is nuciferin and D2 is N-nornuciferin.

### Identification of active hydroxy groups of the aporphine framework

Prior to screening and obtaining all synthesizable derivatives, we identified the position of the active hydroxy group of the aporphine framework that contributes to the antioxidant activity or anti-cell death effect. Apomorphine has two hydroxy groups (-OH) at positions 10 and 11 of its aporphine framework, so to verify which has the essential anti-cell death effect, we produced three similar derivatives: D29, D30, and D31. D29 is a precursor of apomorphine in the synthesis of apomorphine using o-silyl aryl triflate; however, its cytotoxic activity against cancer cells has not been reported^13^.

**Figure 2:**
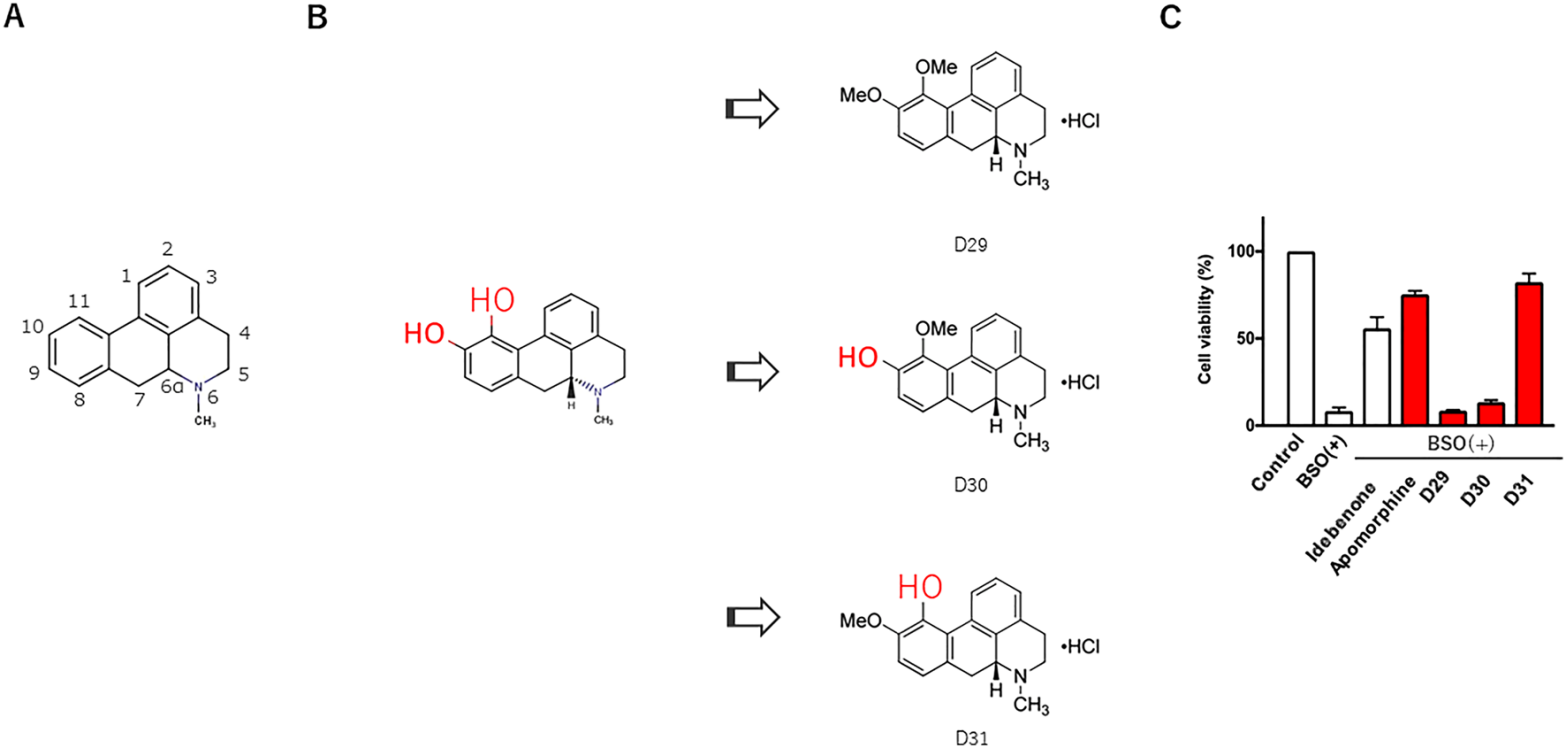
Aporphine framework and identification of active hydroxy groups. (**A**) Aporphine framework. (**B**) Apomorphine has two hydroxy groups at positions 10 and 11. We synthesized three derivatives: D29, D30 and D31. D30 retains a hydroxy group at position 10 and D31 at position 11. (**C**) Among the three derivatives, D31 was the only compound with the same anti-BSO-induced cell death activity as apomorphine.

D31 exhibited a methoxy group (-OMe) at position 10 and a hydroxy group at position 11 in the aporphine alkaloid framework. D31 was the only compound that retained its anti-BSO cell death effect (Fig. 2). Based on this result, we hypothesized that apomorphine derivatives with a hydroxy group at position 11 of the aporphine framework would retain the anti-cell death effect.

### Synthesis of 20 apomorphine derivatives

**Figure 3:**
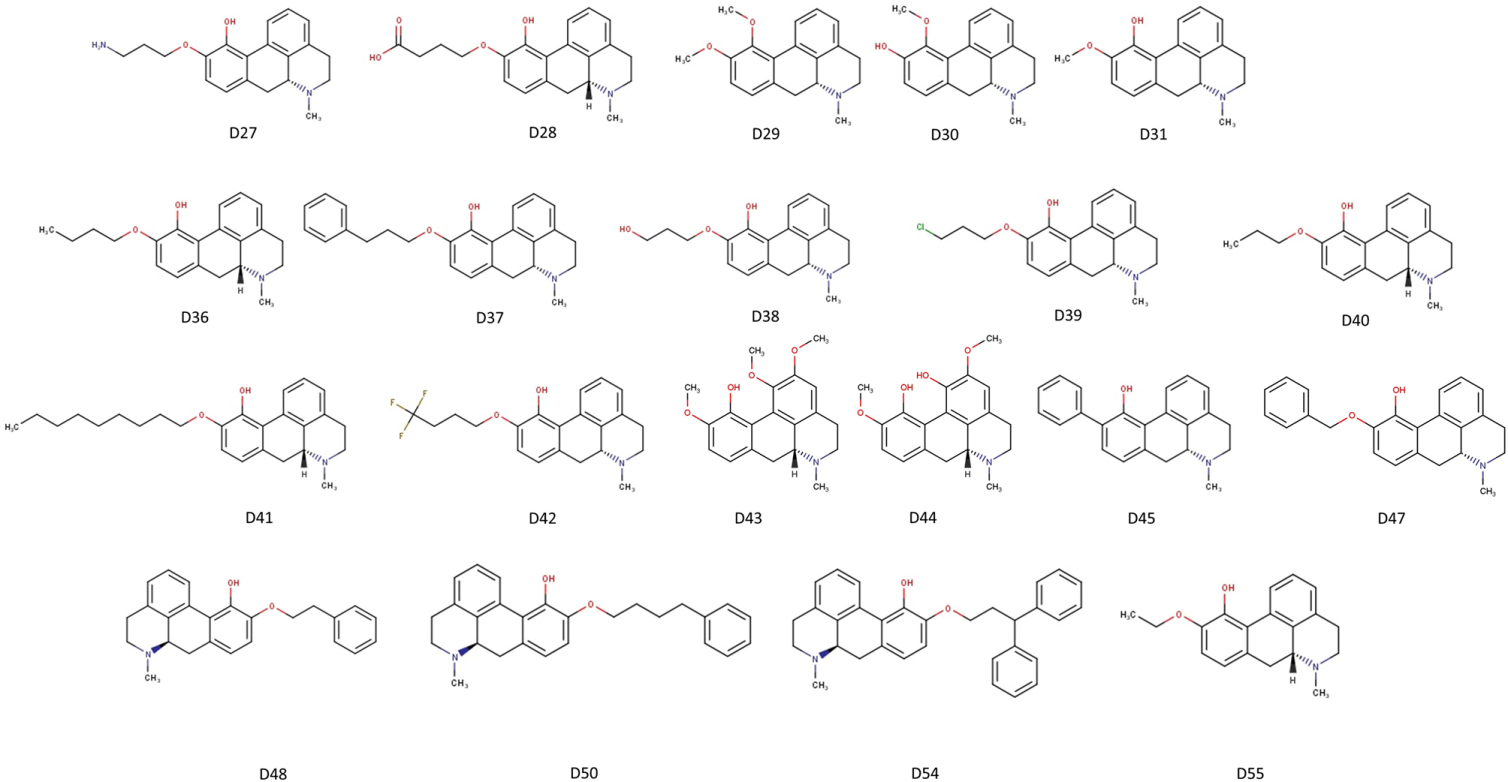
Structural formulae of 20 apomorphine derivatives. The synthesized apomorphine derivatives retained the hydroxy group at position 11 of the aporphine framework, except for D29 and D30.

We synthesized 17 additional apomorphine derivatives in addition to D29, D30, and D31 (Fig. 3). The derivatives retained hydroxy group at position 11 in the aporphine framework. The IUPAC names are listed in Supplementary Table S2. We obtained the D44 structural formula, but it could not be synthesized. The compound is known as corytuberine^14^. D6 was obtained as apocodeine hydrochloride, which we subsequently analyzed and found to be apomorphine. D6 is vacant in this study.

### Dopamine D2 receptor agonist assay

**Figure 4:**
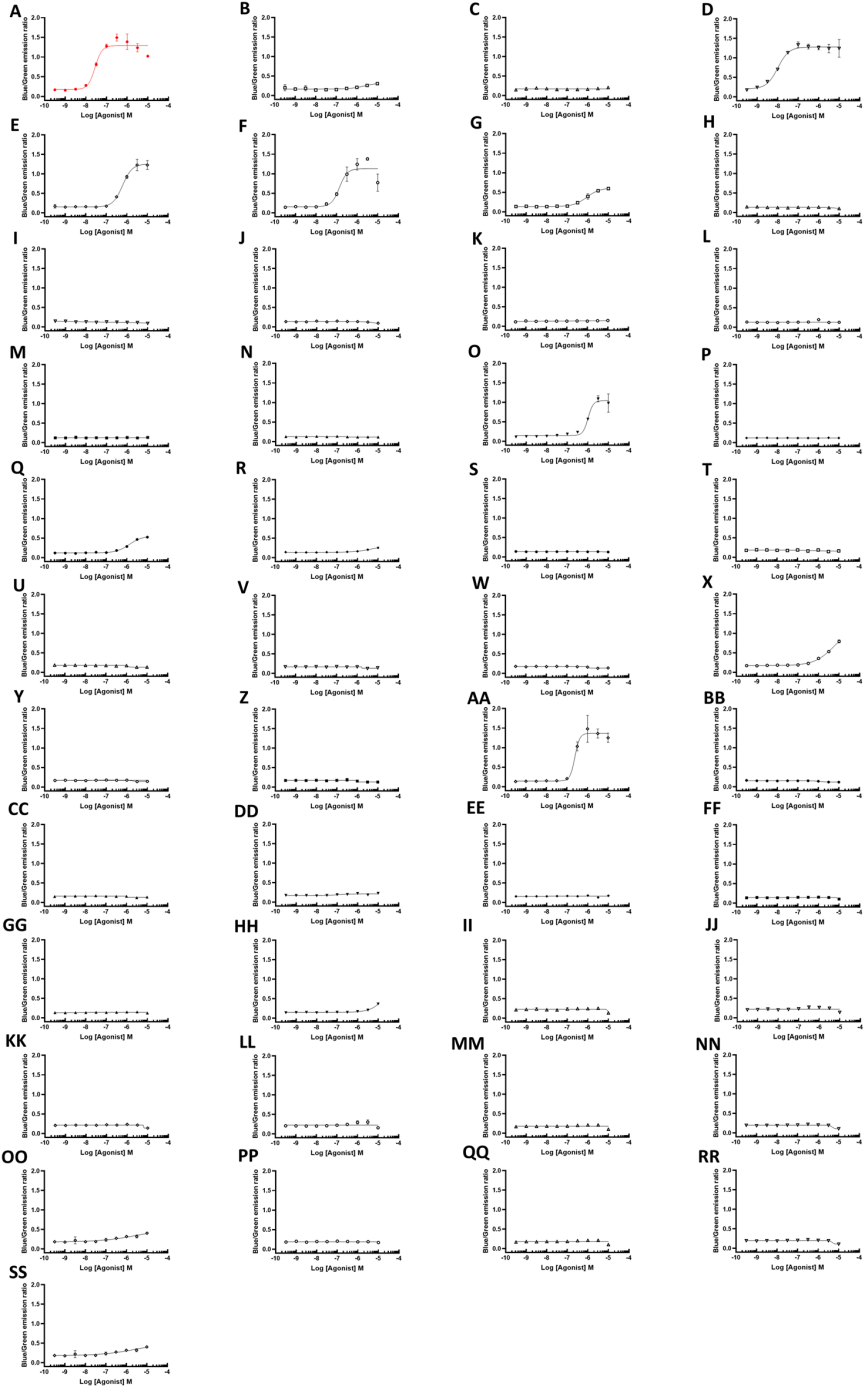
Dopamine D2 receptor activities of apomorphine analogs/derivatives. Using CHO cells that stable express dopamine D2 receptors, dopamine agonist (DA) activities were evaluated and EC50 values were calculated. NA: no activity (**A**) Apo, EC50 = 35.1 nM; (**B**) D1, NA; (**C**) D2, EC50 = NA; (**D**) D3, EC50 = 10.4 nM; (**E**) D4, EC50 = 582 nM; (**F**) D5, EC50 = 206 nM; (**G**) D7, EC50 = 809 nM; (**H**) D8, NA; (**I**) D9, NA; (**J**) D10, NA**; **(**K**) D11, NA; (**L**) D12, NA; (**M**) D13, NA; (**N**) D14, NA; (**O**) D15, EC50 = 935 nM; (**P**) D16, NA; (**Q**) D17, EC50 = 1040 nM; (**R**) D18, NA; (**S**) D19, NA; (**T**) D20, NA; (**U**) D21, NA; (**V**) D22, NA; (**W**) D23, NA; (**X**) D24, EC50 = 1955 nM; (**Y**) D25, NA; (**Z**) D26, NA; (**AA**) D27, EC50 = 254 nM; (**BB**) D28, NA; (**CC**) D29, NA; (**DD**) D30, NA; (**EE**) D31, NA; (**FF**) D36, NA; (**GG**) D37, NA; (**HH**) D38, EC50 = 3651 nM; (**II**) D39, NA; (**JJ**) D40, NA; (**KK**) D41, NA; (**LL**) D42, NA; (**MM**) D43, NA; (**NN**) D45, EC50 = 2634 nM; (**OO**) D47, NA; (**PP**) D48, NA; (**QQ**) D50, NA; (**RR**) D54, NA; (**SS**) D55, NA.

The dopamine D2 receptor agonist activity, which is responsible for emetic side effects, was measured for all apomorphine analogs and derivatives. The compounds D3 and D5 showed half maximal effective concentration (EC50) values of 10.4 and 206 nM, respectively, and were excluded for their high D2 agonist effects that can cause emesis as a side effect (Fig. 4).

### Anti-BSO-induced cell death effect

**Figure 5:**
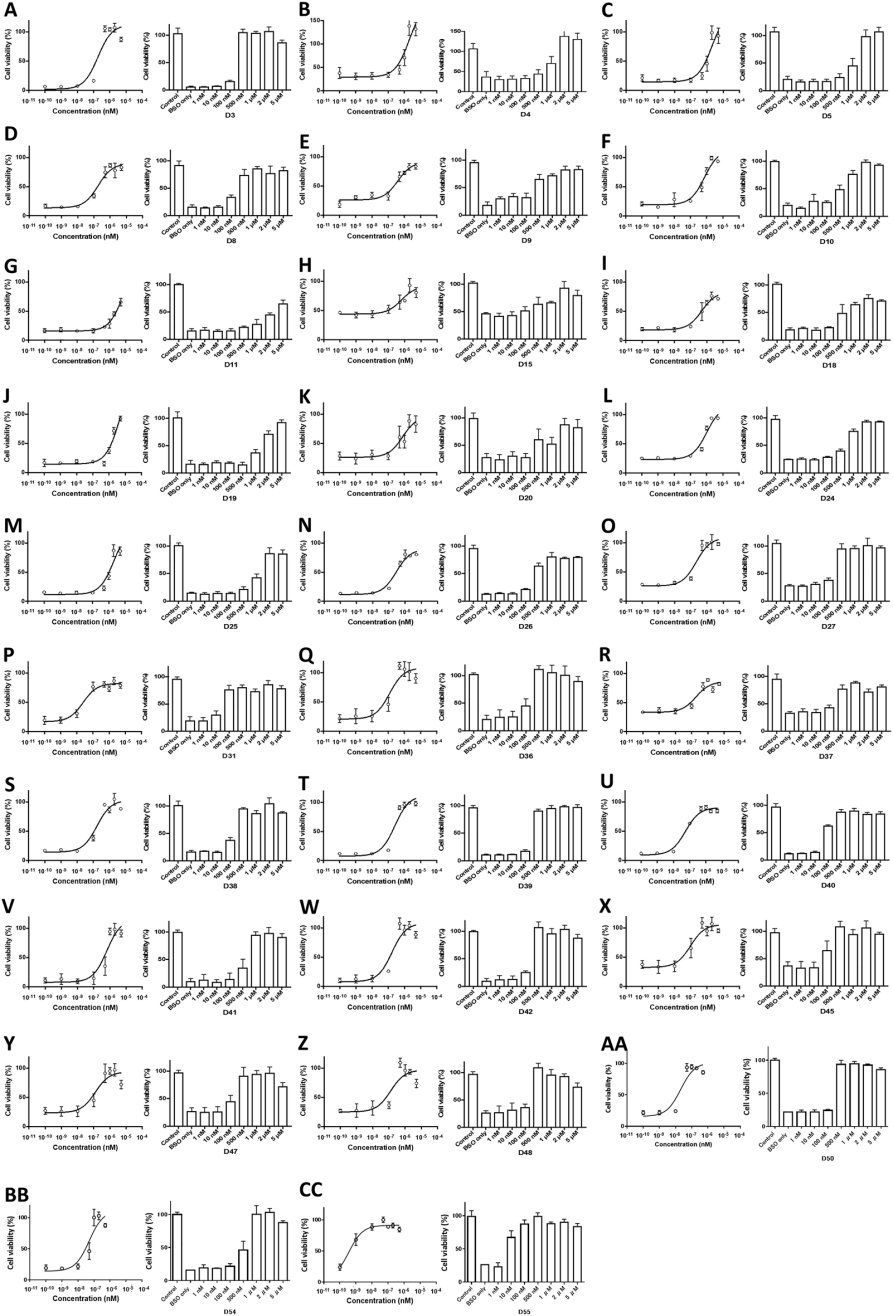
Anti-BSO-induced cell death effect of apomorphine analogs/derivatives. For compounds that showed > 50% cell viability in the BSO-coadded assay, the EC50 of each compound was determined by BSO addition at a concentration of 10%-20% cell viability. (**A)** D3, EC50 = 186 nM; (**B**) D4, EC50 = 1990 nM; (**C**) D5, EC50 = 2110 nM; (**D**) D8, EC50 = 180 nM; (**E**) D9, EC50 = 393 nM; (**F**) D10, EC50 = 750 nM; (**G**) D11, EC50 = 6920 nM; (**H**) D15, EC50 = 613 nM; (**I**) D18, EC50 = 492 nM; (**J**) D19, EC50 = 5180 nM; (**K**) D20, EC50 = 798 nM; (**L**) D24, EC50 = 1026 nM; (**M**) D25, EC50 = 2049 nM; (**N**) D26, EC50 = 289 nM; (**O**) D27, EC50 = 221 nM; (**P**) D31, EC50 = 24 nM; (**Q**) D36, EC50 = 123 nM; (**R**) D37, EC50 = 186 nM; (**S**) D38, EC50 = 165 nM; (**T**) D39, EC50 = 259 nM; (**U**) D40, EC50 = 55 nM; (**V**) D41, EC50 = 648 nM; (**W**) D42, EC50 = 167 nM; (**X**) D45, EC50 = 96 nM; (**Y**) D47, EC50 = 124 nM; (**Z**) D48, EC50 = 128 nM; (**AA**) D50, EC50 = 207 nM; (**BB**) D54, EC50 = 497 nM; (**CC**) D55, EC50 = 4 nM.

The 46 compounds obtained were screened for their anti-BSO-induced cell death effect, with a high viable cell ratio of > 50% noted when co-loaded (1 µM) with BSO (Supplementary Fig. S1). EC50 was measured for the compounds with high viable cell rates of > 50% and for compounds D45-D55 (Fig. 5). D6, D31, D40, D45 and D55 showed particularly high anti-cell death activities with EC50 of < 100 nM. D6 and D31 were synthesized as the same compound, a known apocodeine, while D30 was an isoapocodeine, and D6 was found to be an apomorphine. They were all similar in structures: D31 has a methoxy group, D40 has a propoxy group, D45 has a phenolic group, and D55 has an ethoxy group at position 11 of the aporphine framework (Fig. 3).

### Anti-RSL3-induced cell death effect of apo analogs and derivatives

**Figure 6:**
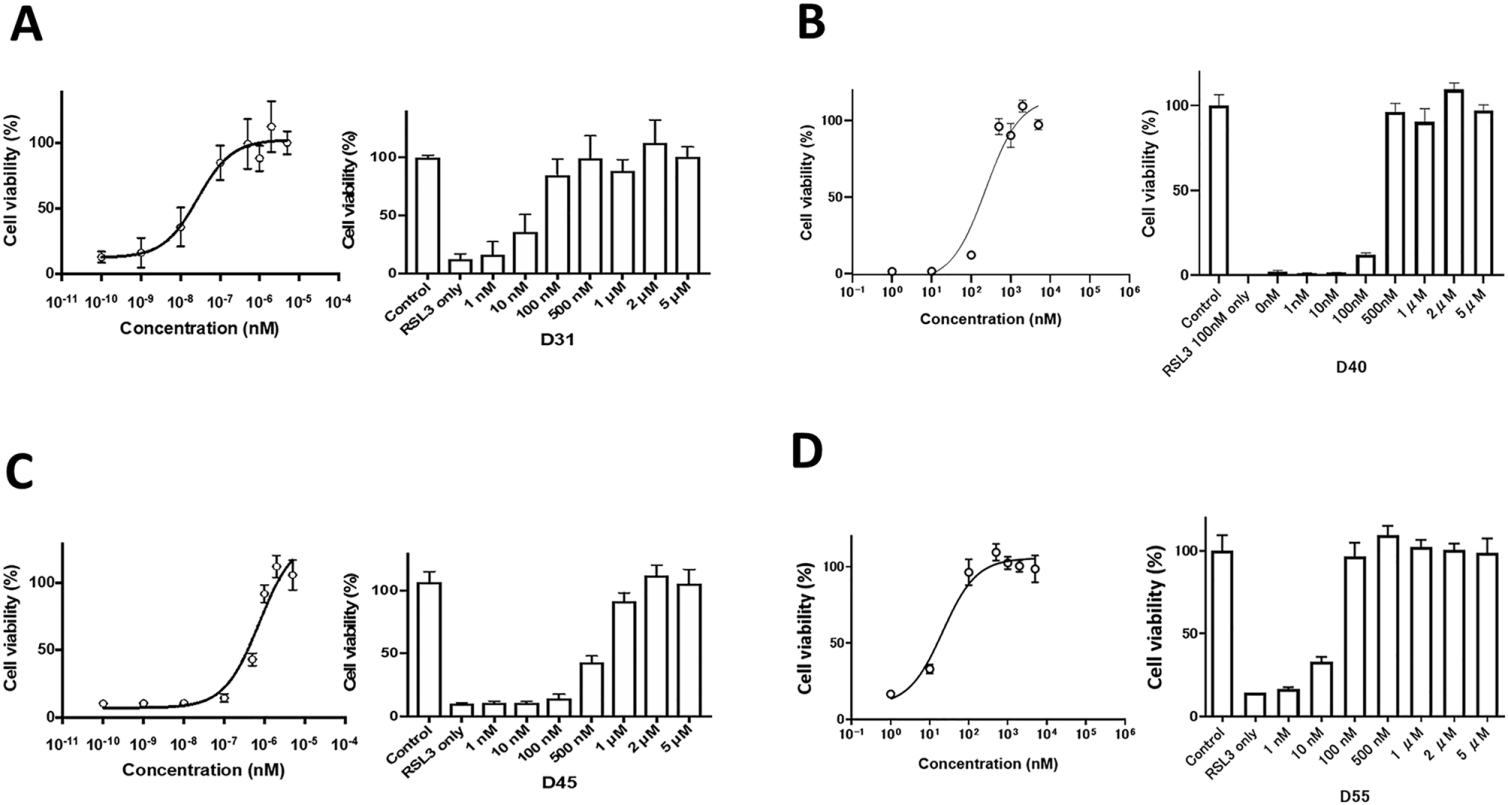
Anti-RSL3-induced cell death effect of apomorphine analogs/derivatives. For four compounds that showed EC50 of < 100 nM the BSO-coadded assay, the EC50 of each compound was determined by RSL3 addition at a concentration of 10%-20% cell viability. D31, D55, D40 have methoxy, ethoxy, and propoxy groups as side chains at position 10 of the aporphine framework, respectively. D31 with a methoxy group and D55 with an ethoxy group showed particularly high anti-RSL3-induced cell death activity. (**A**) D31, EC50 = 27 nM; (**B**) D40, EC50 = 231 nM; (**C**) D45, EC50 = 745 nM; (**D**) D55, EC50 = 21 nM.

We measured the anti-RSL3-cell death activity of above four compounds, D31, D40, D45, and D55 (Fig. 6). None of the compounds exhibited the activity at the dopamine D2 receptors. However, they demonstrated efficacy against RSL3-induced ferroptosis. The anti-ferroptotic activities of the four candidate compounds were evaluated, showing the following results: D31 with an EC50 of 27 nM, D40 with an EC50 of 231 nM, D45 with an EC50 of 745 nM, and D55 with an EC50 of 21 nM. As D45 showed the lower anti-RSL3 cell death activity, we tested the effect on ATP production of D31, 40, and 55.

### Effects of three candidate compounds on mitochondrial ATP production on LS patient-derived skin fibroblasts

**Figure 7:**
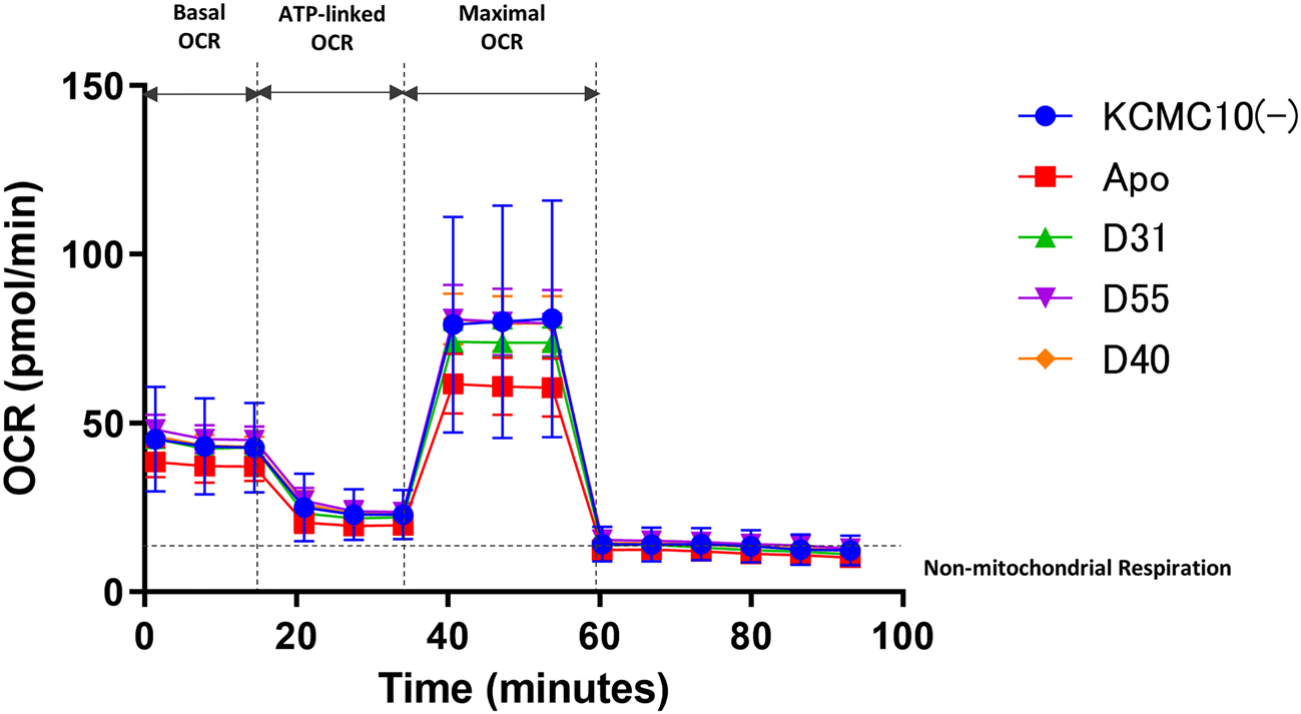
ATP production capacity of LS patient-derived skin fibroblasts. No significant enhancement of ATP production upon administration of apomorphine, D31, D55, or D40.

Apomorphine has been reported to increase ATP production in LS patient-derived skin fibroblasts^9^, but neither D31, D55, nor D40 showed any ability to enhance mitochondrial ATP production in LS patient-derived skin fibroblasts (Fig. 7).

### Inhibitory effects of three candidate compounds on growth differentiation factor 15 (GDF-15) elevation in LS patient-derived dermal fibroblasts

**Figure 8:**
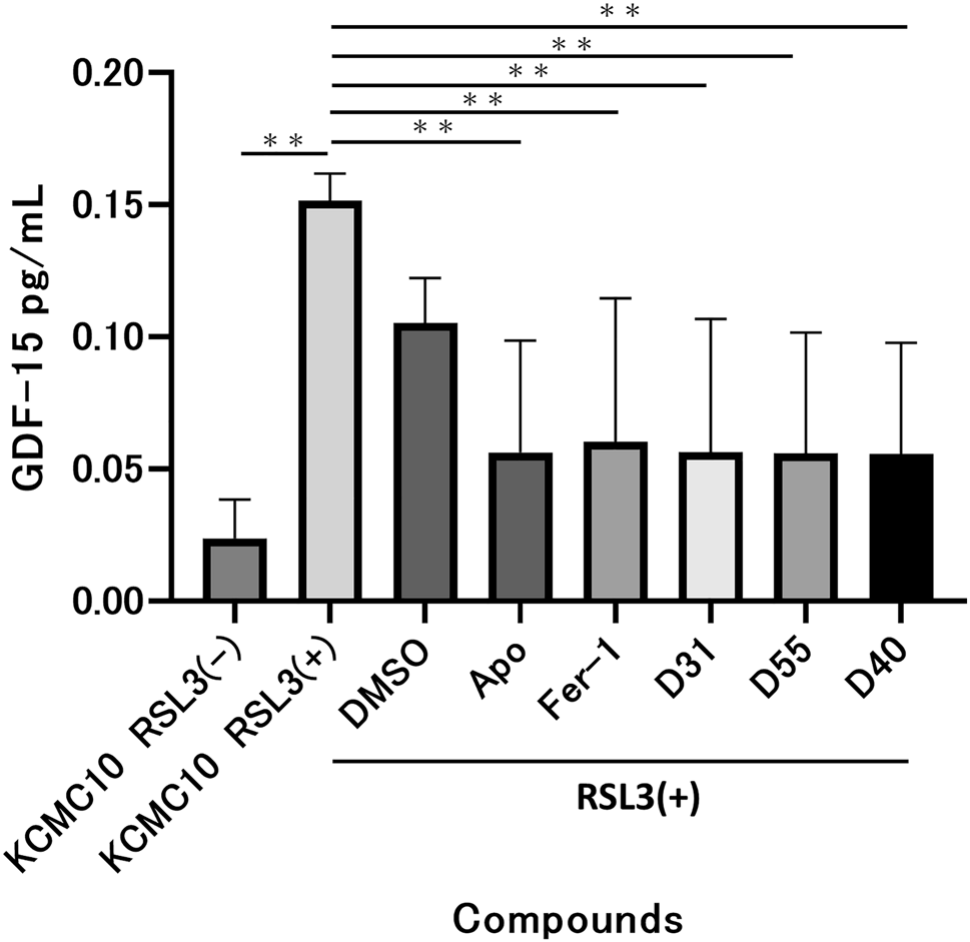
GDF-15 suppression in RSL3 loaded LS fibroblasts of D31, D55, and D40. Compared to the RSL3-only, the groups co-administered with apomorphine, ferrostatin-1, D31, D55, and D40 showed a significant decrease in the concentration of GDF-15 in cell supernatant of LS patient-derived skin fibroblasts.

D31, D55, and D40 suppressed GDF-15 elevation in LS patient-derived skin fibroblasts 24 h after co-administration with RSL3 (Fig. 8). This result was similar to that observed when cell death was induced with BSO^9^.

## Discussion

Apomorphine is a dibenzoquinoline aporphine alkaloid and aporphine alkaloids are compounds that possess an aporphine framework and bioactivity. Many natural aporphine alkaloids have been isolated from several plants in nature, and multiple synthetic pathways have been identified^15^. Natural and synthetic aporphine alkaloids exhibit various pharmacological activities, including antidiabetic, anticancer, and anti-inflammatory effects^16^.

Apomorphine and its derivatives show anti-cell death activity, and the inhibition of ferroptosis is one of their actions^10^. In the present study, we found that apomorphine analogs and derivatives that lack the DA activity but retain anti-cell death effect against BSO or RSL3 on LS patient-derived skin fibroblasts.

We obtained as many apomorphine analogs and derivatives as possible and compared their anti-cell death effects under conditions of oxidative stress. The compounds with high anti-cell death activity had reactive groups at specific positions in the aporphine framework.

Apomorphine was first synthesized in 1845 and many studies have focused on apomorphine for its central nervous system effects have been reported such as sleep dysfunction, apathy, fatigue, attention deficit, dementia, and so on^11,17^. In 1884, its efficacy in patients with PD was hypothesized, and 100 years later, in 1954, its efficacy in PD was reported^11^. Due to its poor bioavailability and short biological half-life, levodopa, an oral dopamine precursor, have appeared in clinical setting^17,18^. Apomorphine was later approved for treating the “off” symptoms of advanced PD in 1987^19^, and then approved in Japan specifically in 2012.

Apomorphine is structurally similar to dopamine and, thus, has broad-spectrum dopamine agonist activity. The polycyclic and amine structures of apomorphine make it highly lipophilic and allow it to easily penetrate the blood–brain barrier^11^. Apomorphine concentrations in the brain may be up to three-fold higher than those in plasma^20^. It and other aporphine alkaloids are known to have diverse pharmacological actions, not only at dopamine receptors, but also at serotonin, adrenergic alpha, and acetylcholine receptors^11,21^. Furthermore, apomorphine has been reported to exert antioxidant activity^21,22^, even before the discovery of ferroptosis^23^, a form of cell death caused by lipid peroxidation.

The mechanism of the antioxidant effect of apomorphine has been discussed and dopamine D4 receptor agonist activity is a candidate^24^. However, we recently reported that the anti-cell death effect of apomorphine is due to its anti-ferroptotic effect, regardless of its dopamine receptor agonist activities^10^, and we found that the synthetic aporphine alkaloids also possess anti-ferroptotic activity. Bulbocapnine, an aporphine alkaloid with a hydroxy group at position 11, has been reported to have in vitro CNS activity^21^ along with nuciferine and several other naturally occurring aporphine alkaloids, and nuciferin has been reported to exert anti-ferroptotic activity^25^.

The strong antioxidant, or anti-ferroptotic effect, of apomorphine and its derivatives is due to the activity of the hydroxy group at position 11 of the aporphine framework, and modification of the functional group at position 10 or other sites may alter membrane permeability and intracellular availability, resulting in an inhibitory effect on cell death. The hydroxy and N-alkyl group at position 11 of aporphine also alter dopamine receptor selectivity. Substitution of the N-methyl group and acylation of the carbon at position 10 have been reported to enhance dopamine D1 receptor affinity^25^. The dopamine receptor affinities of synthetic aporphine alkaloids should be investigated in the future. The catechol groups at positions 10 and 11 of aporphine are responsible for the in vivo clearance and low bioavailability, and the pharmacokinetics may differ for the aporphine alkaloids found in this research^21^.

In our compound synthesis and exploration, we first took the approach to discover the pharmacophore involved in the anti-cell death activity. The results showed that the OH group at position 11 of the aporphine framework is essential for anti-cell death effect and also contributes to DA activity, while the modified group at position 10 reduces DA activity but retains the anti-cell death effect of the OH group at position 11, indicating that the anti-cell death effect increases or decreases with the functional group. The candidate compounds all retained the OH group at position 11. We succeeded in eliminating DA activity while retaining the anti-cell death effect as originally intended.

Apomorphine inhibits GDF-15 elevation in the culture supernatant of LS patient-derived skin fibroblasts^9^, and the three candidate aporphine alkaloids also inhibited GDF-15 elevation. GDF-15 is listed as a biomarker for mitochondrial diseases and is considered a useful endpoint for future in vivo evaluation^26^.

The brain pathology in LS patients shows white matter vacuolization, increased vascularity and gliosis, microglial expansion, and increased IL-6 levels in the cerebellum and brainstem in NDUFS4^-/-^ mice, and neuroinflammation is indicated in LS patients’ brains^27^. The antioxidative and anti-ferroptotic effects of apomorphine and its derivatives need to be evaluated for pathological alterations when administered to the brain tissue.

In the current study, we aimed to eliminate the DA effect from apomorphine, and confirmed that the derivatives have the same antioxidant effect as apomorphine. In the future, it will be necessary to investigate the antioxidant capacity of apomorphine and the derivatives using other methods. The three candidate compounds found in the apomorphine derivatives synthesized in this study will be evaluated in vivo for their pharmacokinetics and pathophysiological effects to determine the final candidate drugs. Moreover, their high anti- ferroptotic activity have potential as a therapeutic agent for diseases related to ferroptosis.

## Subjects and methods

### LS patient-derived skin fibroblasts

The patient with LS was confirmed to have clinical symptoms, biochemical test findings, and pathogenic gene variant for LS among those attending the Department of Pediatrics of the Jichi Medical University Hospital and Kanagawa Children’s Medical Center. Sample collection was approved by the Ethics Committee of Jichi Medical University. Written informed consent was obtained from the patient’s parents. All research was performed in accordance with relevant guidelines and regulations.

A series of experiments was performed using skin fibroblasts established from patients with LS (m.10158 T > C [p.Ser34Pro]; ND3) due to pathogenic mitochondrial gene variants (Supplementary Table S3). ND3 is a component protein of the respiratory chain complex I subunit that is involved in the assembly and activity^28–31^. This patient has been previously reported^32^. Healthy skin fibroblasts purchased from Promo Cell GmbH (#C-12300; Heidelberg, Germany) were used as controls.

### Cell culture

Patient-derived skin fibroblasts were cultured in Dulbecco’s Modified Eagle’s Medium (DMEM) supplemented with 10% (v/v) fetal bovine serum (FBS), 100 units/ml penicillin and 100 μg/ml streptomycin at 37 ℃ in 5% CO_2_. The number of passages of cultured cells ranged from 5 to 15.

### Screening of existing compounds by structural similarity calculation

A total of 26 apomorphine analogs were obtained by structural similarity retrieval out of 2,000,000 commercially available compounds (Akiyama Lab., Tokyo Institute of Technology, Tokyo, Japan). Aporphane, the chemical structure shown in Supplementary Fig. S2 was used as a query and obtained the 26 compounds through publicly available chemical library and database search (Namiki Shoji Co.Ltd., Tokyo, Japan, and Kishida Chemical Co.Ltd., Osaka, Japan). We conducted a substructure search against the compound databases using the aporphane structure as the query. We selected 26 compounds out of 89 hits by the substructure search. Twenty apomorphine derivatives were synthesized (Tokyo Chemical Industry, Co., Ltd., Tokyo, Japan). Supplementary Figure S3-6 shows synthesis of D40 and D55 and H-NMR data among the compounds.

### Dopamine D2 receptor agonist assay

A total of 46 apomorphine analogs and derivatives were screened for dopamine D2 receptor activity using the GeneBLAzer^™^D2-Gqo5 CHO-K1 DA (Invitrogen, Carlsbad, CA, USA) and D2-Gqo5-NFAT-bla CHO-K1 Cell-based Assay (ThermoFischer Scientific, Waltham, MA, USA). CHO-K1 cells were spread in 384-well plates at 10,000 cells/32 μL/well, taking care not to touch the bottom of the wells. Cell-free-wells were injected with the same volume (32 μL) of assay medium. The plates were then incubated in a CO_2_ incubator for 16–20 h. Agonist (apomorphine) and test compounds were then step-diluted using 96-well plates and diluted to 50 Μm–1.58 nM in assay medium (+ 0.5% dimethyl sulfoxide [DMSO]). The diluted solution (8 μL/well) was added to 384-well plates seeded with CHO-K1 cells and incubated in a CO_2_ incubator for 5 h. Substrate Mix (8 μL/well) was added according to instructions and incubated for 2 h at room temperature while light-shielded, followed by measurement using EnVision (PerkinElmer, Waltham, MA, USA) under the following conditions: Scan1 Ex 409/20 nM, Em 460/40 nM, Scan2 Ex 409/20 nM, Em 530/30 nM. The addition of the drug induces D2 receptor activation, which activates signaling pathways and transcription factors, resulting in the expression of a reporter gene (β-lactamase) and cleavage of the fluorescence resonance energy transfer (FRET) substrate, causing the fluorescence to change from green to blue.

### Anti-BSO-induced cell death activity assay

A total of 46 apomorphine (Apo) analogs and derivatives were screened for their anti-cell death activity. BSO inhibits intracellular γ-glutamyl-l-cysteine (GCS). EC50 is the concentration at which a drug exhibits 50% of its minimum to maximum value and is generally used as an indicator of a drug’s efficacy^33^.

LS patient-derived skin fibroblasts for the study were seeded in 96-well plates for cell culture at a concentration of 5000 cells/well and 100 μL medium (10% FBS + DMEM) /well and cultured in a CO_2_ incubator at 37 °C and 5% CO_2_ for 24 h. After 24 h of incubation, reagents were added to the BSO (−) Apo analog/derivative (−) and BSO ( +) Apo analog/derivative ( +) groups; the BSO ( +) Apo ( +) and BSO ( +) ferrostatin-1 ( +) groups were thus established and compared as compounds with known anti-cell death activity. Apomorphine and ferrostatin-1 were both used at a final concentration of 1 μM, and the final concentration of BSO was 500 μM–2 mM. Ferrostatin-1 is a ferroptosis inhibitor that specifically inhibits RSL3-induced cell death in vitro^23,34^. The percentage of viable cells was measured 48 h after addition of each reagent. The absorbance of the generated formazan was measured using a Benchmark Plus microplate reader (Bio-Rad, Hercules, CA, USA). The viable cell rate was measured at each concentration (0, 1,10, 100, 500, 1000, 2000, and 5000 nM) of the Apomorphine analogs/derivatives co-added with BSO and the EC50 was then calculated. Experiments for each compound were performed in at least duplicates.

### Identification of the hydroxy group (-OH) required for anti-cell death activity

Compounds with a structure similar to that of apomorphine were tested for their anti-BSO-induced cell death activity. We identified the hydroxy group required for retaining anti-cell death activity and synthesized 17 additional apomorphine derivatives with a hydroxy group at the same site.

### Anti-RSL3-induced cell death activity assay

Among the apomorphine analogs and derivatives, three with active hydroxy groups required for the anti-cell death effect were extracted and evaluated for anti-RSL3-induced cell death. RSL3 is a glutathione peroxidase 4 (GPx4) inhibitor that specifically induces ferroptosis^35^. Ferroptosis is an iron-dependent non-apoptotic type of cell death caused by lipid peroxidation^23^.

LS patient-derived skin fibroblasts for the study were seeded in 96-well plates for cell culture at a concentration of 5000 cells/well and 100 μL medium (10% FBS + DMEM) /well and cultured in a CO_2_ incubator at 37 ℃ and 5% CO_2_ for 24 h. After 24 h of incubation, the reagents were added to the RSL3 (−) derivative (−) and RSL3 ( +) derivative ( +) groups. The RSL3 ( +) Apo ( +) and RSL3 ( +) ferrostatin-1 ( +) groups were used as controls. The final concentration of RSL3 was 50–200 nM that would cause approximately 80–90% cell death after 24 h of incubation. The percentage of viable cells was measured 24 h after addition of each reagent. The absorbance of the generated formazan was measured with a Benchmark Plus microplate reader (Bio-Rad, Hercules, CA, USA). The viable cell fraction was measured at each concentration (0, 1,10, 100, 500, 1000, 2000, and 5000 nM) of the Apo derivatives co-added with RSL3, and the EC50 was then calculated. Experiments for each compound were performed in at least duplicates.

### Measurement of mitochondrial ATP production capacity

Intracellular metabolic indices that can be evaluated using an extracellular flux analyzer are decreased in LS patient-derived skin fibroblasts, but it has been reported that the addition of apomorphine increases the mitochondrial ATP production capacity^9^. Using the same LS patient-derived skin fibroblasts, we evaluated the mitochondrial function when the target compound, an apomorphine derivative that retains anti-cell death activity, was added. The extracellular flux analyzer Seahorse XFe96 (Agilent Technologies, Santa Clara, CA, USA) was used to evaluate the mitochondrial function. The extracellular flux analyzer measures the oxygen consumption rate (OCR) in the mitochondria during ATP synthesis and can also evaluate the intracellular mitochondrial function. The endpoints were the basal OCR, ATP-linked OCR, maximal OCR, and spare capacity, which are key indicators of the mitochondrial function in assays using living cells^9^. Each indicator was calculated by continuously adding ATP synthase inhibitors (oligomycin, rotenone and antimycin A) and deconjugating agents (carbonyl cyanide-p-trifluoromethoxyphenylhydrazone [FCCP]) during the measurement (Fig. 6). The measurement procedure is as follows: LS patient-derived skin fibroblasts were seeded in 96-well plates for cell culture at a concentration of 20,000 cells/well and 80 μL/well and then incubated in a CO_2_ incubator at 37 ℃ and 5% CO_2_ for 24 h. After 24 h of incubation, Apo derivative (100 nM concentration) or 0.1% DMSO as a control was added. The OCR was measured over time by adding oligomycin (2 μM concentration) 20 min after drug addition, FCCP (2 μM concentration) 50 min later, and rotenone and antimycin A (0.5 μM concentration) 80 min later.

### Measurement of GDF-15 levels in the cell supernatant

GDF-15 is a biomarker that is elevated in the blood of patients with mitochondrial diseases and is used for the diagnosis of mitochondrial diseases and determination of therapeutic efficacy^26,36^. It has also been reported to be upregulated in multiple sclerosis, cardiac failure, and liver disease^37–39^. As previously reported^9^, GDF-15 levels were measured by an enzyme-linked immuno sorbent assay (ELISA) using the Quantikin Human GDF-15 ELISA Kit (R&D Systems, Minneapolis, MN, USA). Samples were obtained from anti-RSL3-induced cell death assays with candidate compounds (D31, D55, and D40), and cell supernatants 24 h after reagent addition were used for the assay. Measured values were calculated with reference to internal standards and compared to GDF-15 values.

### Statistical analysis

The GraphPad Prism software program (version 9; GraphPad Software Inc., Boston, MA, USA) was used to for the statistical analyses. Comparisons between two groups of samples were performed using the two-sample *t*-test, and comparisons between multiple groups were performed using a one-way analysis of variance. Results are expressed as the mean ± standard deviation, with *p* values for any test indicated by *p < 0.05 and **p < 0.01 as statistical significance.

### Supplementary Information

Supplementary Table S1.

Supplementary Table S2.

Supplementary Table S3.

Supplementary Legends.

Supplementary Figure S1.

Supplementary Figure S2.

Supplementary Figure S3.

Supplementary Figure S4.

Supplementary Figure S5.

Supplementary Figure S6.

## Data Availability

The datasets used and/or analysed during the current study are available from the corresponding author on reasonable request.
