# Supplementary material for: Synthetic aporphine alkaloids are potential therapeutics for Leigh syndrome
Source: Sci Rep. 2024 May 21;14:11561. doi: 10.1038/s41598-024-62445-w (PMC11109252; doi:10.1038/s41598-024-62445-w)
Supplement: Supplementary file 1 — Supplementary Table S1. [file 41598_2024_62445_MOESM1_ESM.docx]

**Supplementary Table S1. IUPAC notation of apomorphine and the 26 apomorphine analogs.**

| Apo | (9*R*)-10-methyl-10-azatetracyclo[7.7.1.0²,⁷.0¹³,¹⁷]heptadeca-1(17),2,4,6,13,15-hexaene-3,4-diol |
| --- | --- |
| D1 | (9*R*)-15,16-dimethoxy-10-methyl-10-azatetracyclo[7.7.1.0²,⁷.0¹³,¹⁷]heptadeca-1(17),2,4,6,13,15-hexaene |
| D2 | (9*R*)-15,16-dimethoxy-10-azatetracyclo[7.7.1.0²,⁷.0¹³,¹⁷]heptadeca-1(17),2,4,6,13,15-hexaene |
| D3 | (9*R*)-10-propyl-10-azatetracyclo[7.7.1.0²,⁷.0¹³,¹⁷]heptadeca-1(17),2,4,6,13,15-hexaene-3,4-diol |
| D4 | (9*R*)-10-azatetracyclo[7.7.1.0²,⁷.0¹³,¹⁷]heptadeca-1(17),2,4,6,13,15-hexaene-3,4-diol |
| D5 | (12*R*)-13-propyl-4,6-dioxa-13-azapentacyclo[10.7.1.0²,¹⁰.0³,⁷.0¹⁶,²⁰]icosa-1(20),2,7,9,16,18-hexaene |
| D7 | (9*R*)-16-methoxy-10-methyl-10-azatetracyclo[7.7.1.0²,⁷.0¹³,¹⁷]heptadeca-1(17),2,4,6,13,15-hexaen-15-ol |
| D8 | (12*R*)-15,16-dimethoxy-11-methyl-3,5-dioxa-11-azapentacyclo[10.7.1.0²,⁶.0⁸,²⁰.0¹⁴,¹⁹]icosa-1(20),2(6),7,14,16,18-hexaene |
| D9 | (12*R*)-15-methoxy-11-methyl-3,5-dioxa-11-azapentacyclo[10.7.1.0²,⁶.0⁸,²⁰.0¹⁴,¹⁹]icosa-1(20),2(6),7,14,16,18-hexaene |
| D10 | (12*R*)-16-methoxy-11-methyl-3,5-dioxa-11-azapentacyclo[10.7.1.0²,⁶.0⁸,²⁰.0¹⁴,¹⁹]icosa-1(20),2(6),7,14,16,18-hexaen-18-ol |
| D11 | (9*R*)-4,5,15,16-tetramethoxy-10-methyl-10-azatetracyclo[7.7.1.0²,⁷.0¹³,¹⁷]heptadeca-1(17),2,4,6,13,15-hexaene |
| D12 | 16-hydroxy-3,15-dimethoxy-10,10-dimethyl-10-azatetracyclo[7.7.1.0²,⁷.0¹³,¹⁷]heptadeca-1(17),2,4,6,13,15-hexaen-10-ium |
| D13 | 4,5,15,16-tetramethoxy-10,10-dimethyl-10-azatetracyclo[7.7.1.0²,⁷.0¹³,¹⁷]heptadeca-1(17),2,4,6,13,15-hexaen-10-ium |
| D14 | (12*R*)-17-hydroxy-11,11-dimethyl-3,5-dioxa-11-azapentacyclo[10.7.1.0²,⁶.0⁸,²⁰.0¹⁴,¹⁹]icosa-1(20),2(6),7,14,16,18-hexaen-11-ium |
| D15 | (9*R*)-10-methyl-10-azatetracyclo[7.7.1.0²,⁷.0¹³,¹⁷]heptadeca-1(17),2,4,6,13,15-hexaene-3,4-diol |
| D16 | (9*R*)-15,16-dimethoxy-10,10-dimethyl-10-azatetracyclo[7.7.1.0²,⁷.0¹³,¹⁷]heptadeca-1(17),2,4,6,13,15-hexaen-10-ium |
| D17 | (9*R*)-16-methoxy-10-methyl-10-azatetracyclo[7.7.1.0²,⁷.0¹³,¹⁷]heptadeca-1(17),2,4,6,13,15-hexaen-15-ol |
| D18 | (12*S*)-11-methyl-3,5-dioxa-11-azapentacyclo[10.7.1.0²,⁶.0⁸,²⁰.0¹⁴,¹⁹]icosa-1(20),2(6),7,14,16,18-hexaene |
| D19 | (9*R*)-4,16-dimethoxy-10-methyl-10-azatetracyclo[7.7.1.0²,⁷.0¹³,¹⁷]heptadeca-1(17),2,4,6,13,15-hexaene-5,15-diol |
| D20 | 7,16,17-trimethoxy-11-methyl-3,5-dioxa-11-azapentacyclo[10.7.1.0²,⁶.0⁸,²⁰.0¹⁴,¹⁹]icosa-1(20),2(6),7,14,16,18-hexaene |
| D21 | 17-hydroxy-11,11-dimethyl-3,5-dioxa-11-azapentacyclo[10.7.1.0²,⁶.0⁸,²⁰.0¹⁴,¹⁹]icosa-1(20),2(6),7,14(19),15,17-hexaen-11-ium |
| D22 | 16-hydroxy-3,15-dimethoxy-10,10-dimethyl-10-azatetracyclo[7.7.1.0²,⁷.0¹³,¹⁷]heptadeca-1(17),2,4,6,13,15-hexaen-10-ium |
| D23 | 4,5,15,16-tetramethoxy-10,10-dimethyl-10-azatetracyclo[7.7.1.0²,⁷.0¹³,¹⁷]heptadeca-1(17),2,4,6,13,15-hexaen-10-ium |
| D24 | (9*S*)-10-methyl-10-azatetracyclo[7.7.1.0²,⁷.0¹³,¹⁷]heptadeca-1(17),2(7),3,5,13,15-hexaene-3,4-diol |
| D25 | 4,15,16-trimethoxy-10-azatetracyclo[7.7.1.0²,⁷.0¹³,¹⁷]heptadeca-1(17),2(7),3,5,13,15-hexaen-5-ol |
| D26 | (9*R*)-3,4,15-trimethoxy-10-methyl-10-azatetracyclo[7.7.1.0²,⁷.0¹³,¹⁷]heptadeca-1(17),2,4,6,13,15-hexaen-16-ol |
|  |  |
