# Supplementary material for: Synthetic aporphine alkaloids are potential therapeutics for Leigh syndrome
Source: Sci Rep. 2024 May 21;14:11561. doi: 10.1038/s41598-024-62445-w (PMC11109252; doi:10.1038/s41598-024-62445-w)
Supplement: Supplementary file 2 — Supplementary Table S2. [file 41598_2024_62445_MOESM2_ESM.docx]

**Supplementary Table S2. IUPAC notation of the 20 apomorphine derivatives.**

| D27 | (9R)-4-(3-aminopropoxy)-10-methyl-10-azatetracyclo[7.7.1.0²,⁷.0¹³,¹⁷]heptadeca-1(17),2,4,6,13,15-hexaen-3-ol |
| --- | --- |
| D28 | 4-{[(9R)-3-hydroxy-10-methyl-10-azatetracyclo[7.7.1.0²,⁷.0¹³,¹⁷]heptadeca-1(17),2,4,6,13,15-hexaen-4-yl]oxy}butanoic acid |
| D29 | (9R)-3,4-dimethoxy-10-methyl-10-azatetracyclo[7.7.1.0²,⁷.0¹³,¹⁷]heptadeca-1(17),2,4,6,13,15-hexaene |
| D30 | (9R)-3-methoxy-10-methyl-10-azatetracyclo[7.7.1.0²,⁷.0¹³,¹⁷]heptadeca-1(17),2,4,6,13,15-hexaen-4-ol |
| D31 | (9R)-4-methoxy-10-methyl-10-azatetracyclo[7.7.1.0²,⁷.0¹³,¹⁷]heptadeca-1(17),2,4,6,13,15-hexaen-3-ol |
| D36 | (9R)-4-butoxy-10-methyl-10-azatetracyclo[7.7.1.0²,⁷.0¹³,¹⁷]heptadeca-1(17),2,4,6,13,15-hexaen-3-ol |
| D37 | (9R)-10-methyl-4-(3-phenylpropoxy)-10-azatetracyclo[7.7.1.0²,⁷.0¹³,¹⁷]heptadeca-1(17),2,4,6,13,15-hexaen-3-ol |
| D38 | (9R)-4-(3-hydroxypropoxy)-10-methyl-10-azatetracyclo[7.7.1.0²,⁷.0¹³,¹⁷]heptadeca-1(17),2,4,6,13,15-hexaen-3-ol |
| D39 | (9R)-4-(3-chloropropoxy)-10-methyl-10-azatetracyclo[7.7.1.0²,⁷.0¹³,¹⁷]heptadeca-1(17),2,4,6,13,15-hexaen-3-ol |
| D40 | (9R)-10-methyl-4-propoxy-10-azatetracyclo[7.7.1.0²,⁷.0¹³,¹⁷]heptadeca-1(17),2,4,6,13,15-hexaen-3-ol |
| D41 | (9R)-10-methyl-4-(nonyloxy)-10-azatetracyclo[7.7.1.0²,⁷.0¹³,¹⁷]heptadeca-1(17),2,4,6,13,15-hexaen-3-ol |
| D42 | (9R)-10-methyl-4-(4,4,4-trifluorobutoxy)-10-azatetracyclo[7.7.1.0²,⁷.0¹³,¹⁷]heptadeca-1(17),2,4,6,13,15-hexaen-3-ol |
| D43 | (9R)-4,15,16-trimethoxy-10-methyl-10-azatetracyclo[7.7.1.0²,⁷.0¹³,¹⁷]heptadeca-1(17),2,4,6,13,15-hexaen-3-ol |
| D44 | (9R)-4,15-dimethoxy-10-methyl-10-azatetracyclo[7.7.1.0²,⁷.0¹³,¹⁷]heptadeca-1(17),2,4,6,13,15-hexaene-3,16-diol |
| D45 | (9R)-10-methyl-4-phenyl-10-azatetracyclo[7.7.1.0²,⁷.0¹³,¹⁷]heptadeca-1(17),2,4,6,13,15-hexaen-3-ol |
| D47 | (9R)-4-(benzyloxy)-10-methyl-10-azatetracyclo[7.7.1.0²,⁷.0¹³,¹⁷]heptadeca-1(17),2,4,6,13,15-hexaen-3-ol |
| D48 | (9R)-10-methyl-4-(2-phenylethoxy)-10-azatetracyclo[7.7.1.0²,⁷.0¹³,¹⁷]heptadeca-1(17),2,4,6,13,15-hexaen-3-ol |
| D50 | (9R)-10-methyl-4-(4-phenylbutoxy)-10-azatetracyclo[7.7.1.0²,⁷.0¹³,¹⁷]heptadeca-1(17),2,4,6,13,15-hexaen-3-ol |
| D54 | (9R)-4-(3,3-diphenylpropoxy)-10-methyl-10-azatetracyclo[7.7.1.0²,⁷.0¹³,¹⁷]heptadeca-1(17),2,4,6,13,15-hexaen-3-ol |
| D55 | (9R)-4-ethoxy-10-methyl-10-azatetracyclo[7.7.1.0²,⁷.0¹³,¹⁷]heptadeca-1(17),2,4,6,13,15-hexaen-3-ol |
