# Supplementary material for: Synthetic aporphine alkaloids are potential therapeutics for Leigh syndrome
Source: Sci Rep. 2024 May 21;14:11561. doi: 10.1038/s41598-024-62445-w (PMC11109252; doi:10.1038/s41598-024-62445-w)
Supplement: Supplementary file 3 — Supplementary Table S3. [file 41598_2024_62445_MOESM3_ESM.docx]

**Supplementary Table S3. Fibroblast cell line from an LS patient.**

| Cell | Phenotype | Age | Variant | Amino Acid | Protein | Heteroplasmy (%) |
| --- | --- | --- | --- | --- | --- | --- |
| KCMC10 | LS | 0 | m.10158T>C | p.Ser34Pro | ND3 | 90 |

LS: Leigh syndrome.

Respiratory chain activities from fibroblasts were as follows: complex Ⅰ 9.8%, Ⅱ 93.9%, Ⅲ 94.5%, Ⅳ 47.6%, CS 100.9%. The heteroplasmy rate was analyzed by the deep sequencing of the pathogenic region^13^. Fibroblasts were used after fewer than 15 passages.
