# Supplementary material for: Synthetic aporphine alkaloids are potential therapeutics for Leigh syndrome
Source: Sci Rep. 2024 May 21;14:11561. doi: 10.1038/s41598-024-62445-w (PMC11109252; doi:10.1038/s41598-024-62445-w)
Supplement: Supplementary file 4 — Supplementary Legends. [file 41598_2024_62445_MOESM4_ESM.docx]

**Supplementary Figure S1.**

The 45 compounds were then screened for their anti-BSO-induced cell death effect with a high viable cell ratio of >50% noted when co-loaded (1 µM) with BSO. The compounds after D43 were directly evaluated for EC50.

**Supplementary Figure S2.**

Aporphane. The compound used as a query for chemical search.

**Supplementary Figure S3.**

Data upon D31.

**Supplementary Figure S4.**

Data upon D40.

**Supplementary Figure S5.**

Data upon D45.

**Supplementary Figure S6.**

Data upon D55.
