# Supplementary figures and images for: Synthetic aporphine alkaloids are potential therapeutics for Leigh syndrome
Source: Sci Rep. 2024 May 21;14:11561. doi: 10.1038/s41598-024-62445-w (PMC11109252; doi:10.1038/s41598-024-62445-w)

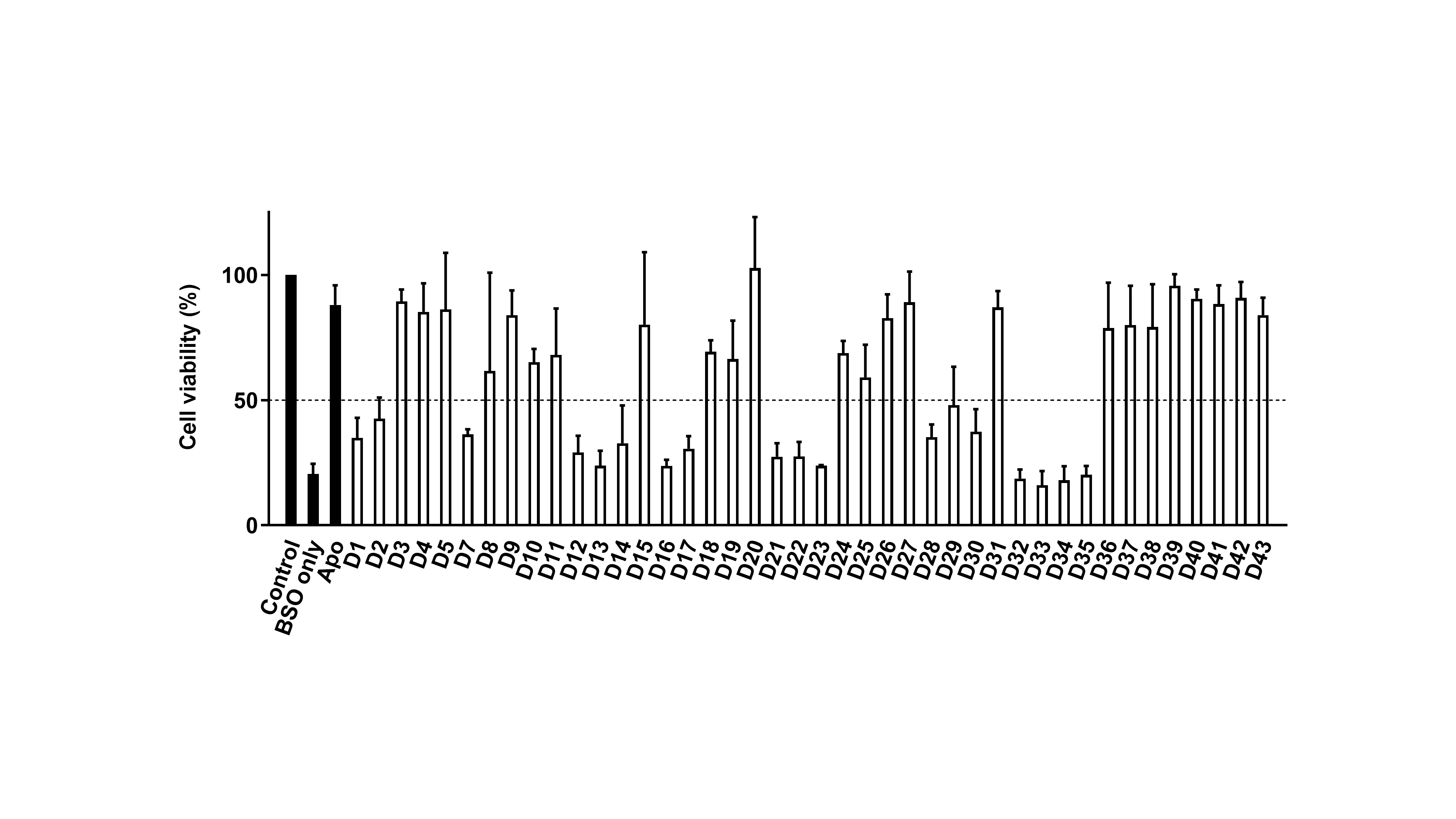

Supplement: Supplementary file 5 — Supplementary Figure S1. [file 41598_2024_62445_MOESM5_ESM.tif]

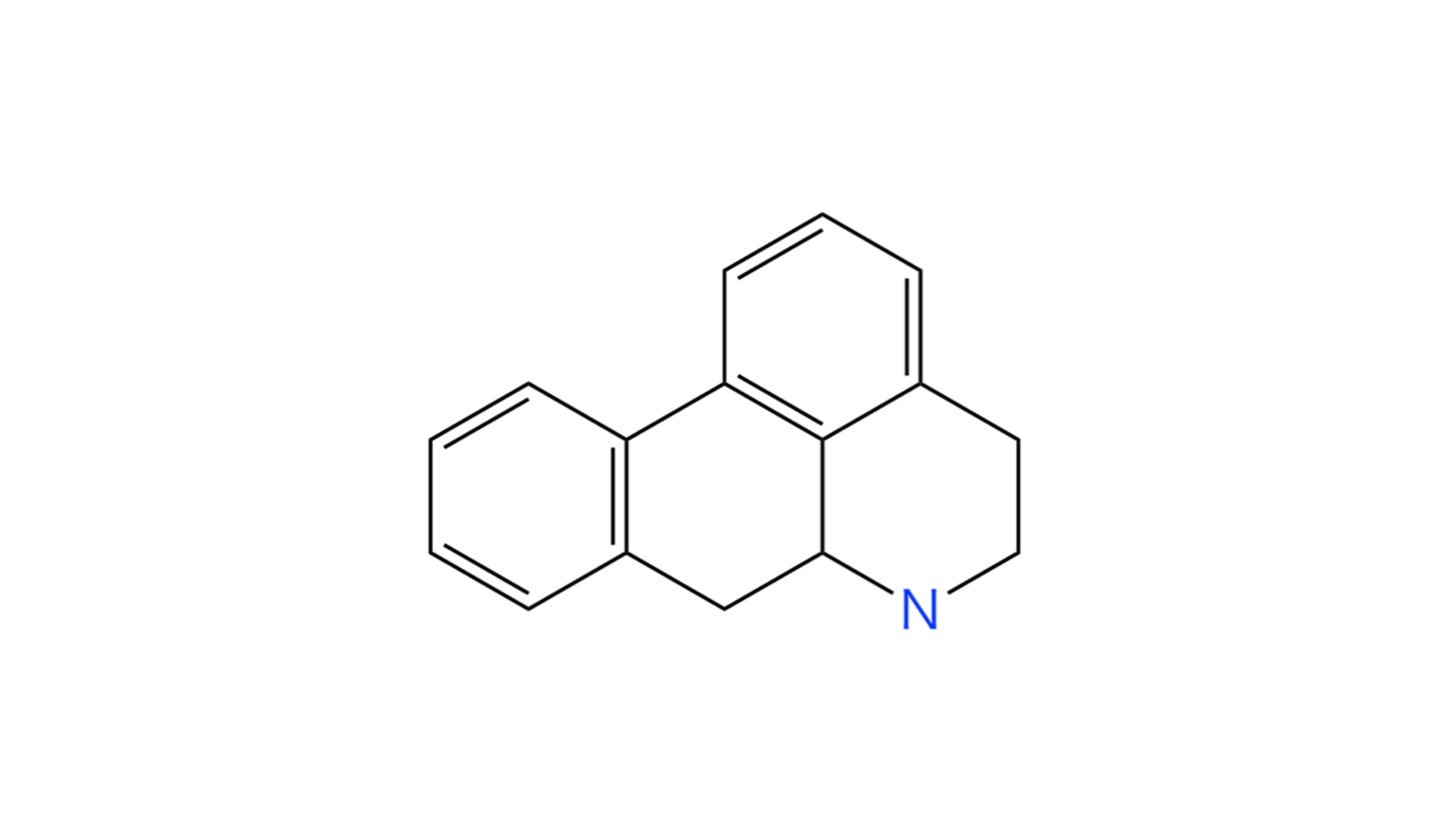

Supplement: Supplementary file 6 — Supplementary Figure S2. [file 41598_2024_62445_MOESM6_ESM.tif]

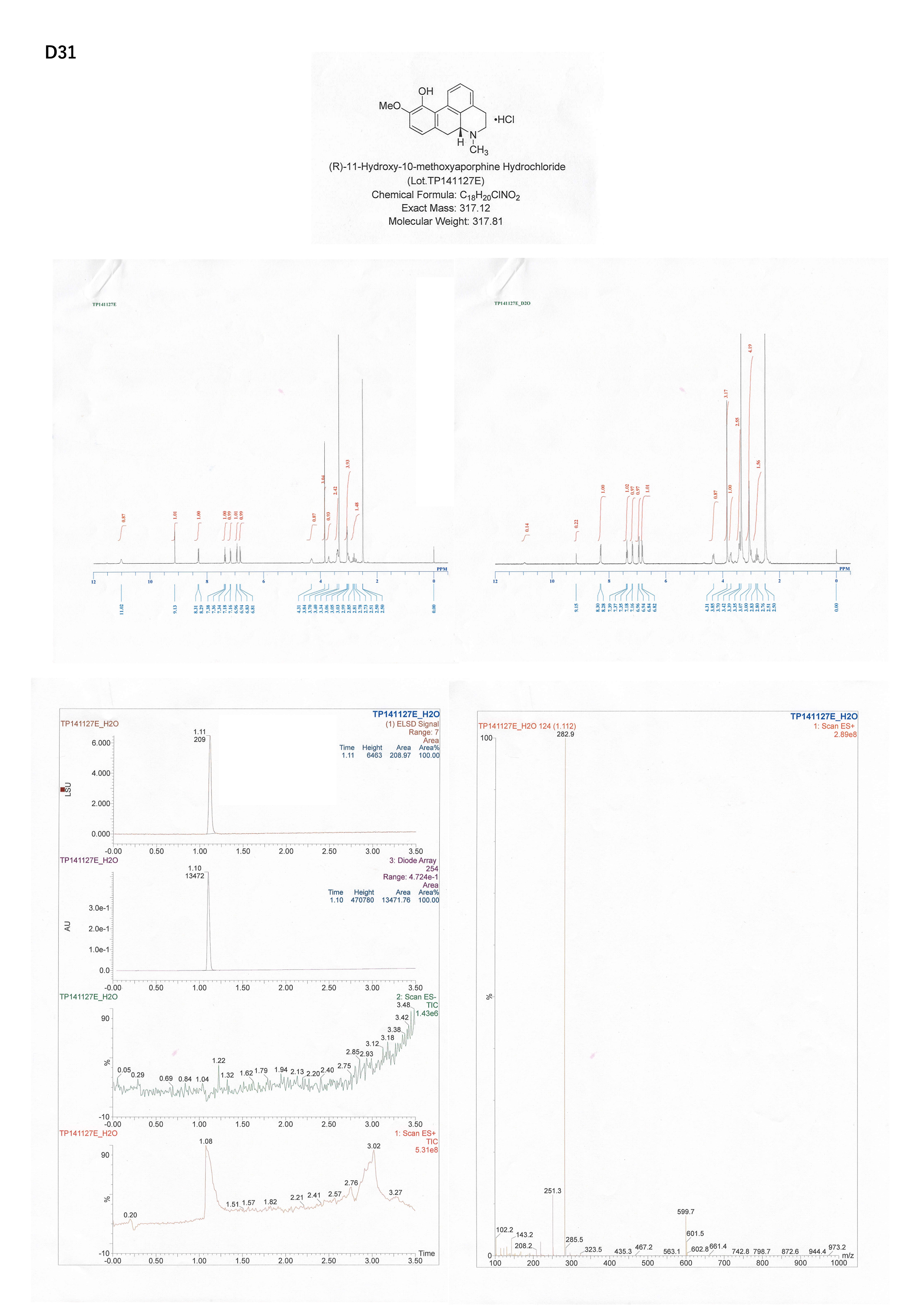

Supplement: Supplementary file 7 — Supplementary Figure S3. [file 41598_2024_62445_MOESM7_ESM.tif]

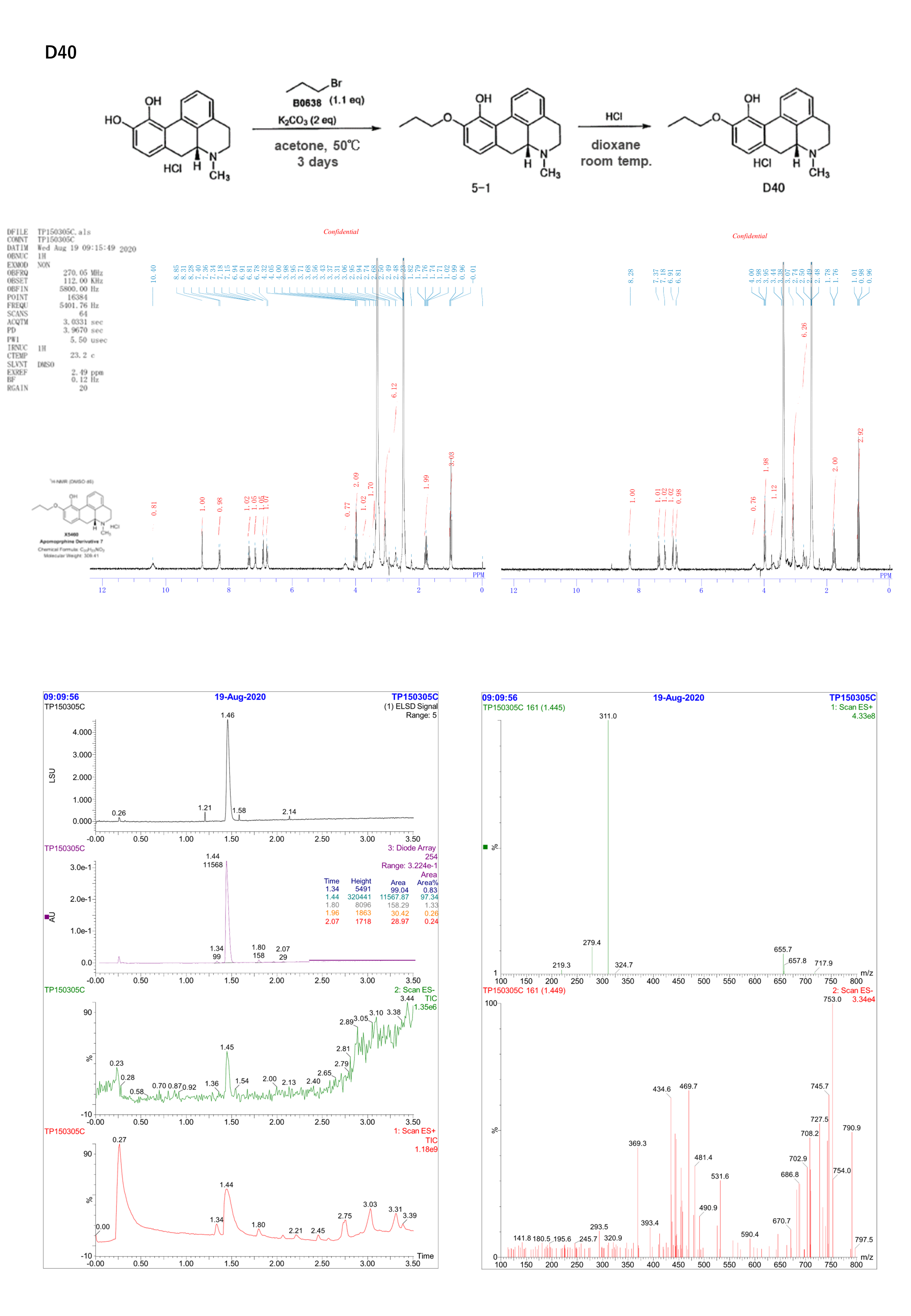

Supplement: Supplementary file 8 — Supplementary Figure S4. [file 41598_2024_62445_MOESM8_ESM.tif]

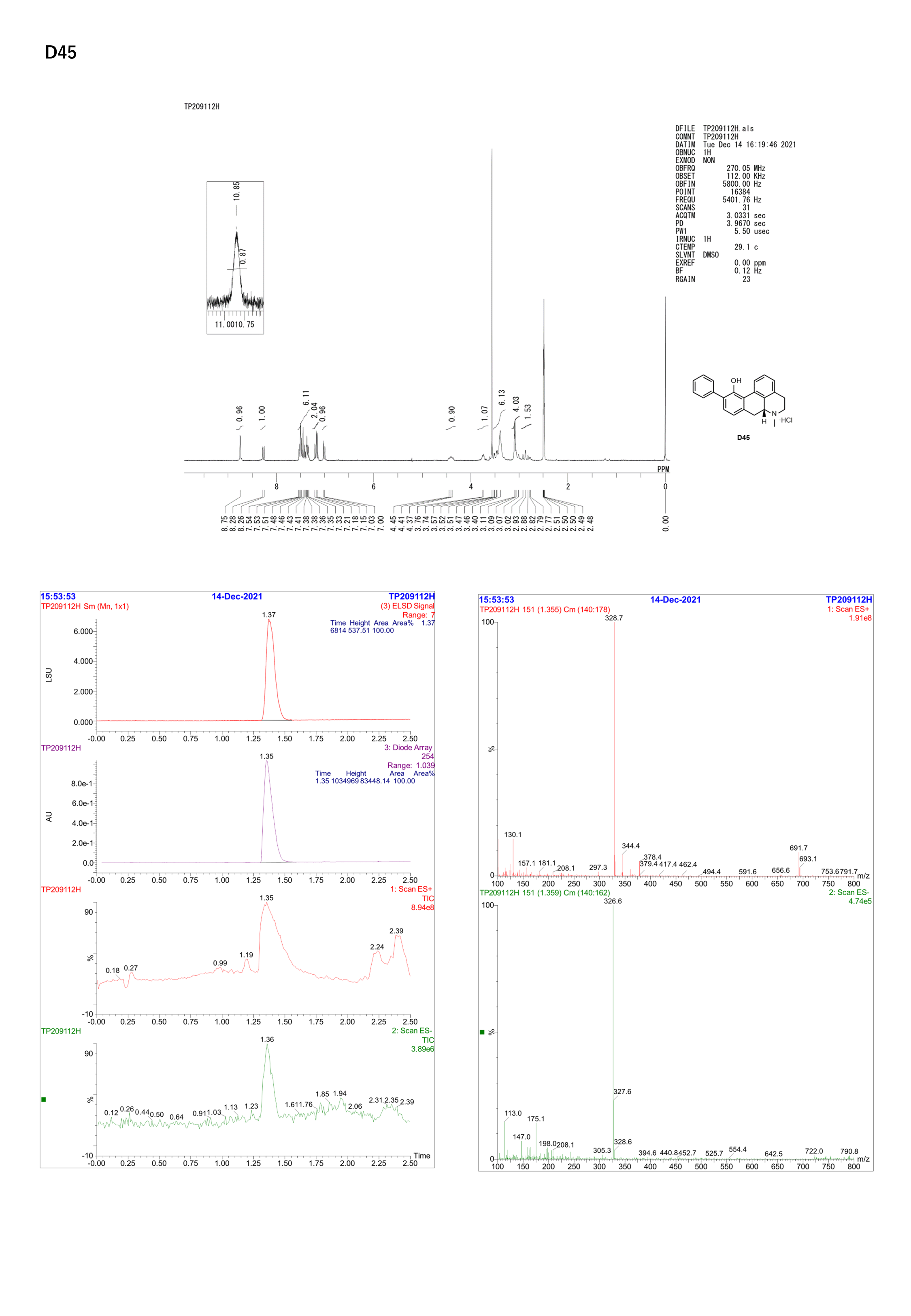

Supplement: Supplementary file 9 — Supplementary Figure S5. [file 41598_2024_62445_MOESM9_ESM.tif]

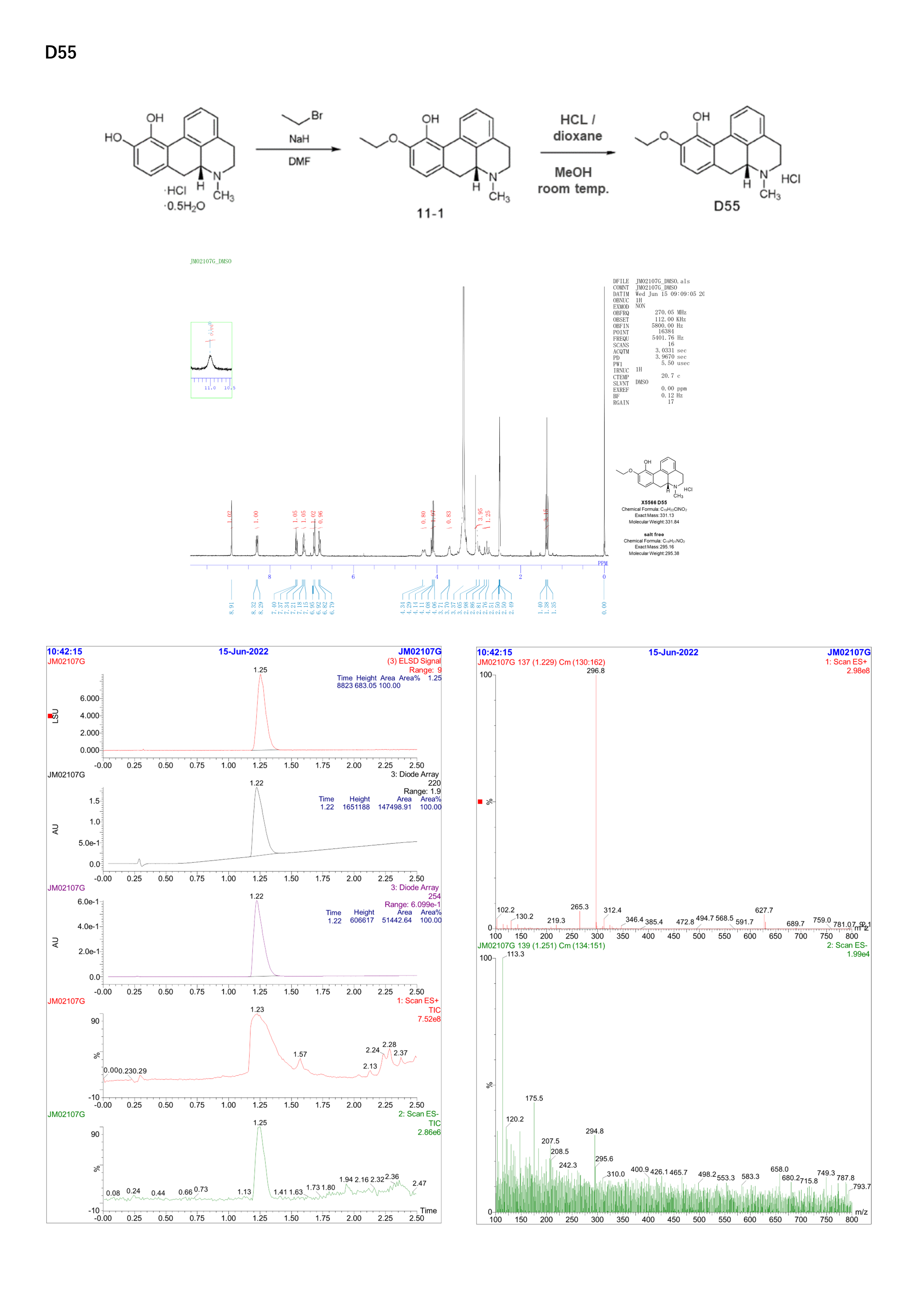

Supplement: Supplementary file 10 — Supplementary Figure S6. [file 41598_2024_62445_MOESM10_ESM.tif]
